# The Incidence, Cost, and Burden of Concussion in Women’s Rugby League and Rugby Union: A Systematic Review and Pooled Analysis

**DOI:** 10.1007/s40279-022-01645-8

**Published:** 2022-02-03

**Authors:** Doug A. King, Patria A. Hume, Karen Hind, Trevor N. Clark, Natalie Hardaker

**Affiliations:** 1 Sports Performance Research Institute New Zealand (SPRINZ), Faculty of Health and Environmental Science, Auckland University of Technology, Auckland, New Zealand; 2 Traumatic Brain Injury Network (TBIN), Auckland University of Technology, Auckland, New Zealand; 3 School of Science and Technology, University of New England, Armidale, NSW Australia; 4 National Institute of Stroke and Applied Neuroscience (NISAN), Faculty of Health and Environmental Science, Auckland University of Technology, Auckland, New Zealand; 5 Wolfson Research Institute for Health and Wellbeing, Department of Sport and Exercise Sciences, Durham University, Durham, UK; 6 Faculty of Sport, Event Management, Tourism and Hospitality, International College of Management Sydney, Manly, NSW Australia; 7 Emergency Department, Hutt Valley District Health Board, Private Bag 31-907, Lower Hutt, New Zealand

## Abstract

**Background:**

The extent of concussion injury in the rugby codes for women is unclear.

**Objective:**

Our aim was to review all published studies reporting concussion injuries from match and training participation in rugby codes and report the pooled data estimates for rugby league and union concussion injury epidemiology.

**Methods:**

We conducted a systematic literature analysis of concussion in rugby league and rugby union for published studies from January 1990 to July 2021. Data from 16 studies meeting the inclusion criteria were extracted for women’s concussion injuries and were subsequently pooled. Costs from Accident Compensation Corporation (ACC) data were attributed to the results to provide cost estimates.

**Results:**

The pooled analysis match injury incidence of women’s concussion was higher for rugby league (10.3 per 1000 match hours) than rugby 15 s (2.8 per 1000 match hours) or rugby 7 s (8.9 per 1000 match hours). There was a fourfold difference in the pooled incidence of concussion in women’s rugby league (risk ratio [RR] 4.53, 95% confidence interval [CI] 1.8–11.3]; *p* = 0.0001) when compared with rugby 15 s. There was also a ninefold higher risk of a concussion during match participation compared with training participation for women’s rugby 15 s (RR 9.3, 95% CI 1.29–66.78; *p* = 0.0070). The total estimated costs for the concussions reported were NZ$1,235,101. For rugby 7 s, the pooled concussive injury burden was 33.2 days.

**Conclusions:**

Our pooled analysis clarified the extent of concussion injury and the possible associated costs at several levels of the game for women’s rugby codes. The pooled mean days lost because of concussions was 33 days. As this was considerably longer than the 7- to 10-day expected timeframe outlined in the Concussion in Sport Consensus statement, these guidelines need to be updated to include sex-specific differences.

**Supplementary Information:**

The online version contains supplementary material available at 10.1007/s40279-022-01645-8.

## Key Points

**Table:** 

Rugby league had higher concussion rates than rugby union for women.
Rugby 7 s had higher concussion rates than rugby 15 s for women.
The higher risk of concussion during matches compared with training varied with participation level.

## Introduction

Rugby union and rugby league are international collision sports played by junior, amateur, semi-professional and professional players worldwide [1–4]. Both have similar characteristics, with forwards and backs players in possession of the ball advancing into the opposition’s territory to score a try by passing the ball backwards or kicking the ball forward in the opposition’s territory. The key differences between league and union are that league has 13 not 15 players on the field; it does not have line outs, rucks or mauls; and players can only hold possession of the ball for a maximum of six tackles before handing the opposition the ball [2]. Rugby league and union, at the senior level of participation, are played on the same size fields, for two 40 min halves with a 10 min break in the middle, although this can vary by age or competition [1, 5]. Although rugby league is typically played with 13 players, rugby union has two versions of participation on the same size field. These are rugby 15 s and the popular shortened version of rugby union, termed rugby 7 s, where seven players compete on a full size rugby field over two 7-min halves [6]. Rugby 7 s matches are typically held during tournaments via a multi-team or multi-game structure over a shortened period (typically 2–3 days) [6].

Female involvement in rugby 15 s and rugby 7 s has increased in popularity, with over 2 million women participating under the same rules as their male counterparts at the community and elite levels of participation [7]. This has been similar in women’s rugby league, with a reported 29% increase in participation numbers reported in Australia and an increased competition pathway in both Australia and England [8]. Despite the increases in participation for both rugby union and rugby league, there are a paucity of studies reporting on match injuries. It has been recently identified [9] that only seven papers have been available since 1990 reporting on women’s rugby match injuries [10–16], compared with more than 113 studies [1, 17, 18] reporting on men’s rugby. One paper has reported on female rugby league [19] participation compared with more than 25 [20–22] studies reporting on male rugby league. This is similar across a variety of sports research, with one study reporting an under-representation of female participant research across three leading sports medicine research journals [23].

Although females participate in match activities under the same rules as males, females reportedly have higher injury risks, even though they have lower physiological indices (e.g. reduced speed and less agility, lower muscular power, lower estimated maximal aerobic power) compared with men [24]. Interestingly, injury patterns also differ between males and females in other sports such as basketball [25], football [26], and handball [27].

In New Zealand, most rugby union and league participants are amateur (i.e., they derive income from another source). This is similar to other league-playing nations. Therefore, the majority of injuries occur at that level of play. Of those papers published reporting on women’s rugby union and league, there is a paucity of studies at the amateur and junior levels of participation in both codes. There is also an assumption that the findings of the epidemiological studies on professional participants translate to other cohorts of participants in other countries but this is yet to be tested [28].

There is a growing body of evidence [29–32] identifying that following sports-related concussion, female athletes experience greater symptom severity and take longer to recover when compared with male athletes. There is likely a combination of intrinsic and extrinsic factors that contribute to this observed sex difference. It has been suggested that it could be due to more reporting of symptoms in females [33, 34], lower neck strength in females [35], sex differences in white matter volume [36, 37], hormonal influence [38, 39], and differences in neural networks utilisation [40]. It has also been reported [41] that the smaller axons in females have a greater probability of microtubule damage compared with males when exposed to equivalent acceleration/deceleration forces. Importantly, data also show that earlier access to medical care can improve time to symptom recovery, especially in females [42]. These factors highlight further opportunity to tailor and improve prevention, care, and recovery of female athletes to ensure a safe and sustained return to sport.

### Objective

The purpose of this pooled analysis was to provide estimates for rugby league and rugby union training and match concussion injury epidemiology for women.

## Methods

The methodology utilised in this pooled analysis was similar to previous pooled analysis studies reporting rugby league injuries [43, 44] and followed the steps as described by Friedenreich [45, 46]. An additional advantage to utilising a pooled analysis approach is that the same statistical model can be utilised with data from methodologically diverse studies [47]. The review was registered with the International Prospective Register of Systematic Reviews (PROSPERO) on 27 January 2020 (Registration No. CRD42020166833). Guidelines for the reporting of systematic reviews (Preferred Reporting Items for Systematic Reviews and Meta-Analyses [PRISMA] [48]) and observational studies (Meta-analysis Of Observational Studies in Epidemiology [MOOSE] [49]) were followed. The PRISMA and MOOSE guidelines contain checklists that were utilised for conducting and reviewing the included studies.

### Search Strategy for Identification of Databases

Articles were identified from an initial search of the online databases from January 1990 to July 2021. The search was undertaken using the key search terms of ‘Rugby Union’, Rugby League’, ‘rugby’, ‘union’, ‘league’ and ‘football’ AND ‘women’, ‘female’, ‘woman’, ‘females’, AND ‘match’ OR ‘training’ as well as the injury terms ‘athletic injuries’, ‘concussion’, ‘sports concussion’, ‘sports-related concussion’, ‘brain concussion’, ‘brain injury’, ‘brain injuries’, ‘mild traumatic brain injury’, ‘mild TBI’, ‘mTBI’, ‘traumatic brain injury’, ‘TBI’, ‘craniocerebral trauma’, ‘head injury’, and ‘brain damage’. The reference lists of those articles retrieved for inclusion in this review were also hand searched to identify any other relevant articles. Key articles were retrieved via online databases and through hand searching reference lists, and these were also used for further searches using the Web of Science Cited Reference function. During the second stage of the literature search, the titles and abstracts of articles were reviewed to assess eligibility for inclusion in this review.

### Inclusion/Exclusion Criteria

To establish some control over the heterogeneity of the different studies [49], inclusion criteria were established. Published studies that reported the incidence of injury in rugby league and rugby union match and training activities were collated and included in the pooled analysis if they:

1. were available in English; and
2. were published in a peer reviewed journal or book; and
3. were prospective cohort studies; and
4. reported the match or training time exposure enabling the calculation of women’s rugby 15 s, rugby 7 s, rugby league player time injury rates; and
5. reported concussions as a result of match or training injuries.

Studies were excluded from this review if it was identified that the publication:

1. was unavailable in English; or
2. did not provide match or training exposure enabling the calculation of player time rates; or
3. did not report on concussions that occurred because of match or training activities; or
4. combined male and female sex match or training exposure and did not differentiate; or
5. was a case study; or
6. utilised previously published data on concussions; or
7. was a meta-analysis or systematic review of rugby league and rugby union injuries.

All references were downloaded into a dedicated EndNote library (Endnote, X9). The library was reviewed, and duplicate records were identified and removed. All publications identified were initially screened by publication title and abstract to identify eligibility. The full-text versions of the remaining articles were then retrieved and evaluated against the inclusion criteria. Those studies meeting the inclusion criteria were included in the review. Searches were limited to ‘English language’ only. The references of all relevant articles were searched for further articles. In cases of discrepancies of eligibility, another author assessed the publication to screen for eligibility.

### Procedures

All the studies included in the pooled analysis were observational in design. Two authors extracted the study characteristics and numerical data and assessed the quality by adhering to the protocol for systematic review of observational studies (MOOSE) [49] [see electronic supplementary Table S1]. This approach enabled a more precise estimate of the effects of influential factors and took into account confounding factors (participation level and age) and the heterogeneity of the studies [47].

### Assessment of Publication Quality

All included studies were independently assessed by two authors reporting on the article quality utilising the STrengthening the Reporting of OBservational studies in Epidemiology (STROBE**)** statement [50]. The statement provides a 22-item checklist guidance on the reporting of observational studies in order to facilitate a critical appraisal of the study and for the interpretation of the results. Following the appraisal, the included studies were categorised as either poor, moderate or good quality based on the percentage of fulfilled items on the STROBE checklist, with cut-off values of < 50, 50–80 and > 80%, respectively [51].

### Data Extraction

Those studies meeting the inclusion criteria underwent data extraction for information pertaining to level of participation, concussion and injury definition utilised, reported concussions and player position/group. Not all studies reported the same information in relationship to injury incidence or number of concussive injuries recorded.

### Reconciliation of Incidence Data and Injury Costs

Not all studies included in the systematic review and pooled analysis utilised the standardised method for injury reporting (i.e., per 1000 h of exposure). As a result, calculations were required to convert some study data to the standardised method for injury reporting to enable pooled analysis to be conducted. The mean costs per concussion injury have been reported in several studies [52–56]. Only two of these studies have specifically reported on the associated costs of women participants [52, 53] but these were from two different reporting periods and had a wide variance in mean costs per concussion injury. More recently, a study reported 10 years of sports-related concussion costs and included female costs per concussion for moderate-to-severe claims (MSCs). This study was utilised to calculate the approximate associated costs for concussion across all the studies included in this review. The reported mean costs for concussions from this study were adjusted for inflation (Reserve Bank inflation adjustor; https://www.rbnz.govt.nz/monetary-policy/inflation-calculator) to 2021 values (rugby union: $10,741; rugby league: $12,002) and calculated per reported concussion.

### Injury Definitions

To enable meaningful comparisons, the sports injury definitions of the included studies were categorised into broad groups [57]. The definition of an injury is a contentious issue [58, 59] and there have been many variations on what constitutes a recordable injury. Although there are two broadly accepted definitions (medical treatment and loss-of-time-fully-inclusive) there are advantages and disadvantages to these definitions [57]. The definitions utilised are dependent on what the authors are reporting, such as injuries that result in missed match participation only (semi-inclusive time loss), missed match and training (full-inclusive) or an all-inclusive injury definition [57]. The results of these different definitions is that some papers will report some injuries (time loss in match and training activities) or eliminate them if they did not result on time loss from matches [57, 60]. For the purpose of this review, these groups consisted of: (1) *medical attention/treatment injury* (any injury that requires the assistance of sports medicine personnel with or without time loss from training or completion); (2) *full-inclusive time-loss injury* (any injury that results in time lost from competition and/or training); (3) *semi-inclusive time-loss injury* (any injury that results in time lost from competition only); and (4) *all-inclusive injury* (an injury that requires the assistance of sports medicine personnel and/or that results in time loss from competition and/or training) [60]. All included studies were reviewed to identify any concussion definitions utilised.

### Pooled Analysis of Concussion Incidence

A pooled analysis of the included studies was undertaken where homogeneity occurred in terms of the injury definition utilised and the reporting of injury incidence was per 1000 match or training hours. This strategy has been previously utilised in rugby 15 s [9, 18, 61] and rugby league [22, 43, 44] epidemiological studies to combine the information provided into a single estimate [46, 62]. By pooling the data, the information provided can then be statistically reanalysed, providing more precise injury data [46].

### Statistical Analyses

A combined estimate of injuries within a specific sport through pooled analysis [63] provides more precise evidence and meaningful information about the sport, while controlling for between-study variation due to individual subcohort characteristics [46]. Data from the individual studies were combined. Incidence rates and 95% confidence intervals (CIs) were calculated [64] where data were available and were reported according to the methodology utilised in the individual studies. Reviewer inter-rater reliability was assessed utilising the Cohen’s Kappa (*κ*) statistic. The level of agreement was categorised as none, minimal, weak, moderate, strong and almost perfect, with cut-offs of < 0.20, 0.21–0.39, 0.40–0.59, 0.60–0.79, 0.80–0.90, and > 0.90, respectively [65]. The pooled calculation of the incidence of concussion was undertaken to report the incidence per 1000 h and the 95% CIs. To compare between injury rates, risk ratios (RRs) were used. To test for significant differences between studies and player positions, Chi-square goodness-of-fit tests were utilised. All statistics were carried out using the SPSS statistical software packages (IBM SPSS Statistics for Windows, Version 22.0, Armonk, NY: IBM Corp).

## Results

**Fig. 1:**
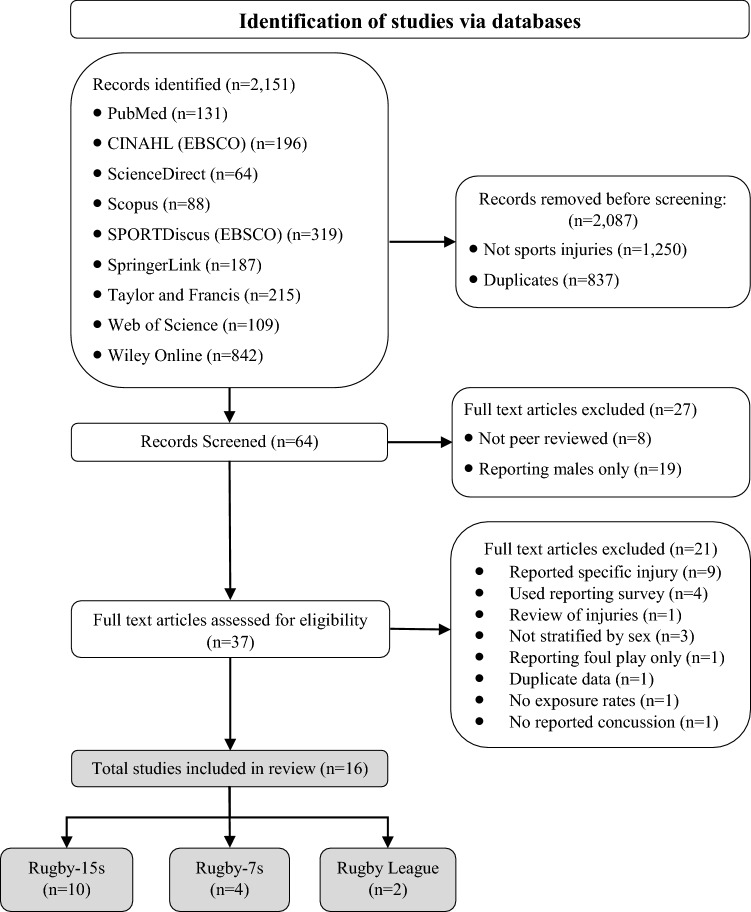
PRISMA 2020 [48] flow diagram for the identification, screening, eligibility, and inclusion of studies for the pooled analysis of match and training women’s rugby league and rugby union concussion injuries. *PRISMA* Preferred Reporting Items for Systematic Reviews and Meta-Analyses

Sixteen studies [10, 11, 13–16, 19, 66–74] met the inclusion criteria for this review (see Fig. 1). Nine of the included studies [10, 11, 13–16, 70, 73, 74] reported on women’s rugby 15 s match injuries, four studies [66–68, 72] reported on women’s rugby 7 s, two studies [19, 69] reported on women’s rugby league, and two studies [14, 71] reported on women’s rugby 15 s training injuries. Although included in the review, some studies were excluded from the pooled analysis. One of these studies [15] combined both rugby 15 s and rugby 7 s, while another study [16] only reported the number of concussions. Three studies [14, 15, 73] utilised the athlete exposures (AEs) rate and two of these three studies [15, 73] reported on match injuries. Other studies were reviewed but were excluded as they were not peer reviewed [75–77], reported on a specific injury [67, 78–85], did not stratify the results by sex [86–90], did not report concussions [12] or exposure rates [91], assessed injuries as a result of foul play [92], utilised a self-reporting survey [87, 92, 93], reported on the costs [52–55, 94, 95], reported previously included data [6, 67, 68, 96–99], or were reviews [1, 3, 4, 9, 100–102]. Reviewer inter-rater reliability was assessed as strong (*κ* = 0.84; *p* < 0.0001) between two of the reviewers. All the studies included in this review were considered to be of moderate quality [51].

More than half of the studies (62.6%) utilised either an all-inclusive [10, 13–15, 68] or fully-inclusive [11, 66, 67, 70, 71] injury definition. Upon pooling the data, the all-inclusive injury definition resulted in a concussion incidence of 1.0 (95% CI 0.7–1.4) per 1000 match hours for rugby 15 s, whereas the fully-inclusive injury definition resulted in a concussion incidence of 3.6 (95% CI 1.4–9.7) per 1000 match hours. For rugby 7 s, the all-inclusive injury definition resulted in a concussion incidence of 7.7 (95% CI 6.0–9.8) per 1000 match hours, whereas the fully inclusive concussion incidence was 12.7 (95% CI 8.4–19.3) per 1000 match hours. Less than one-fifth (18.8%) of the studies [16, 72, 74] utilised a semi-inclusive injury definition but they did not provide exposure hours and could not be pooled. Two studies [69, 73] did not report an injury definition and one study [19] utilised a medical attention/treatment injury definition. Although all the studies did report on concussion, only two studies [15, 70] provided a definition for the identification of a concussive injury.

The studies reporting rugby 15 s match injury concussion data were drawn from 23,772 match exposure hours (professional: 2300 match hours; amateur/national/collegiate: 21,472 match hours), 7440 player game hours, 76,073 AEs and studies reporting rugby 15 s. Training injury concussion data were drawn from 3339.5 training exposure hours and 23,746 AEs (see Table 1). Studies reporting rugby 7 s match injury concussion data were drawn from 10,705 match hours, and studies reporting on women’s rugby league match injuries were drawn from 484.8 match exposure hours.

**Table 1:** Pooled analysis of concussions in women’s rugby 15 s, rugby 7 s and rugby league for match and training exposure activities according to participation level, by total exposure hours, number of concussions reported and rate per 1000 h or AEs with 95% confidence intervals and estimated costs

Activity and level of participation	Exposure hours	Concussion no	IR (95% CI)	Estimated costs^a^
**Rugby Union**				
** Match concussion injuries reported per 1000 match/player hours**				
*Women's rugby 15 s*				
World Cup				
Schick et al. [10]	1200	4	3.3 (1.3–8.9)^b^	$42,964
Taylor et al. [11]	1100	4	3.6 (1.4–9.7)^b^	$42,964
Pooled World Cup	2300	8	3.5 (1.7–7.0)	$85,928
National				
Carson et al. [13]	1513	4	2.6 (1.0–7.0)^b^	$42,964
Amateur domestic				
King et al. [70]	559	9	16.1 (8.4–31.0)^c,d,e,f^	$96,669
All Ireland League				
Yeomans et al. [74]	3620	20	5.5 (3.1–7.9)^b^	$214,820
Collegiate				
Kerr et al. [14]	15,780	25	1.6 (1.1–2.3)^b^	$268,525
Amateur rugby 15 s pooled	21,472	58	2.1 (1.5–2.8)	$622,978
** Rugby 15 s pooled**	**23,772**	**66**	**2.8 (2.1–3.4)**^**g,h**^	**$708,906**
*Women’s rugby 7 s*				
Amateur/elite candidate				
Lopez et al. [67]	2591	21^†^	8.1 (5.3–12.4)^i,j^	$225,561
Under 19				
Lopez et al. [68]	454	3	6.6 (0.0–14.1)^j^	$32,223
Elite 8-season				
Fuller and Taylor [66]	3938	65	16.5 (12.5–20.5)^j,k^	$698,165
Elite Australian				
Toohey et al. [72]	3722	6	1.6 (0.3–2.9)^i,j,k,l^	$64,446
** Rugby 7 s pooled**	**10,705**	**95**	**8.9 (7.1–10.7)**^**m,n,o**^	**$1,020,395**
**Match concussion injuries reported in another format**				
*Women's rugby 15 s*				
Collegiate				
Langevin et al. [73]	1199 AEs^p^	4^p^	3.3 (0.1–6.6) per 1000 AEs^p^	$42,964
High school				
Collins et al. [16]	NR	11^p^	Unable to calculate	$118,151
*Combination of rugby 15 s and rugby 7 s data*				
Peck et al. [15]	68,633 AEs^p^	30^p^	0.4 (0.3–0.6) per 10,000 AEs^p^	$322,230
**Training concussion injuries reported per 1000 training hours**				
*Women’s rugby 15 s*				
Amateur domestic				
King et al. [71]	3339.50	1	0.3 (0.0–2.1)	$10,741
**Training concussion injuries reported in another format**				
Collegiate				
Kerr et al. [14]	23,746 AEs	7	0.3 (0.1–0.6) per 1000 AEs	$75,187
**Rugby League**				
**Match concussion injuries reported per 1000 match/player hours**				
National tournament				
King and Gabbett [19]	329.2	2	6.1 (1.5–24.3)	$24,004
Amateur club				
King et al. [69]	155.6	3	19.3 (6.2–59.8)	$36,006
**Rugby league pooled**	**484.8**	**5**	**10.3 (4.3–24.8)**^**m,n,o**^	**$60,010**

*IR* incidence rate, *CI* confidence interval, *AEs* athlete exposures, *NR* not reported

^a^Costs are reported in New Zealand dollars (NZ$) based on the average cost per concussion claim [55]

^b^Significant difference (*p* < 0.05) vs. Amateur

^c^Significant difference (*p* < 0.05) vs. National

^d^Significant difference (*p* < 0.05) vs. World Cup

^e^Significant difference (*p* < 0.05) vs. All Ireland League

^f^Significant difference (*p* < 0.05) vs. Collegiate

^g^Significant difference (*p* < 0.05) vs. Rugby 7 s Pooled

^h^Significant difference (*p* < 0.05) vs. Rugby League Pooled

^i^Significant difference (*p* < 0.05) vs. Rugby 7 s Elite 8 Season

^j^Significant difference (*p* < 0.05) vs. Rugby 7 s Australian

^k^Significant difference (*p* < 0.05) vs. Rugby 7 s Amateur/Elite

^l^Significant difference (*p* < 0.05) vs. Rugby 7 s Under 19

^m^Significant difference (*p* < 0.05) vs. Rugby 15 s Pooled World Cup

^n^Significant difference (*p* < 0.05) vs. Rugby 15 s Pooled Amateur

^o^Significant difference (*p* < 0.05) vs. Rugby 15 s Pooled

^p^Combined match and practice exposures

The pooled analysis concussion injury incidence for womens rugby 15 s match injuries was 2.8 (95% CI 2.1–3.4) per 1000 match hours (see Table 1). Although women’s World Cup rugby 15 s recorded a higher pooled concussion incidence rate than amateur rugby 15 s (RR 1.3, 95% CI 0.6–2.7; *p* = 0.5015), this was not significant. Amateur women’s rugby 15 s reported a higher incidence of concussion (16.1 [95% CI 8.4–31.0] per 1000 match hours) than World Cup (RR 4.8, 95% CI 1.5–15.6; *p* = 0.0037), national (RR 6.1, 95% CI 1.9–19.7; *p* = 0.0006) and collegiate (RR 10.2, 95% CI 4.8–21.7; *p* < 0.0001) rugby 15 s levels of participation.

The pooled concussion incidence of rugby 7 s was 8.9 (95% CI 7.1–10.7) per 1000 match hours. Participants at the elite level of women’s rugby 7 s participation recorded a higher concussion incidence (16.5 [95% CI 12.5–20.5] per 1000 match hours) than the amateur/elite candidate (RR 2.0, 95% CI 1.3–3.3; *p* = 0.0038) level of participation. There was a fourfold difference in the pooled incidence of concussion in women’s rugby league (RR 4.53, 95% CI 1.8–11.3; *p* = 0.0001) when compared with women’s rugby 15 s, and a ninefold increase in the risk of concussion in women’s rugby 15 s match activities (RR 9.3, 95% CI 1.29–66.78; *p* = 0.0070) when compared with women’s rugby 15 s training activities. The total estimated costs for the concussions reported was New Zealand dollars (NZ$) $2,358,584. More than one-third (39.4%; NZ$1,020,395) of the estimated total costs were attributed to women’s rugby 7 s, while women’s rugby 15 s total estimated costs were slightly less (27.4%; NZ$708,906).

Only four studies [11, 66–68] reported player position for concussion (see Table 2). Although backs recorded more concussions than forwards in women’s World Cup (RR 0.9, 95% CI 0.1–6.2; *p* = 0.8927) and rugby 7 s (RR 1.0, 95% CI 0.7–1.5; *p* = 0.9279), these differences were not significant. Elite rugby 7 s forwards recorded more concussions (17.2 [95% CI 11.9–24.7] per 1000 match hours) than forwards at the women’s World Cup (RR 5.0, 95% CI 1.2–21.1; *p* = 0.0138) and amateur/elite candidate rugby 7 s participants (RR 2.8, 95% CI 1.2–6.3; *p* = 0.0130). Elite backs at the rugby 7 s level of participation (12.8 [95% CI 9.7–16.8] per 1000 match hours) recorded more concussions than backs at the amateur/elite candidates (RR 4.1, 95% CI 1.0–17.0; *p* = 0.0349) level of participation but this was not significant. Of the studies that reported player positions, backs recorded higher pooled estimated costs for women’s rugby union concussions (NZ$271,572 vs. NZ$210,084).

**Table 2:** Concussions reported for women’s rugby 15 s and rugby 7 s for forwards and backs, by total exposure hours, number of concussions reported and rate per 1000 h or AEs with 95% confidence intervals

	Forwards			Estimated costs^a^	Backs			Estimated costs^a^
	*n*	Hours	IR (95% CI)		*n*	Hours	IR (95% CI)	
Rugby 15 s								
Women’s World Cup								
Taylor et al. [11]	2	587	3.4 (0.9–13.6)^b^	$21,482	2	513	3.9 (1.0–15.6)^b^	$21,482
Rugby 7 s								
Elite 8 season								
Fuller and Taylor [66]	29	1688	17.2 (11.9–24.7)^c,d^	$311,489	36	2250	16.0 (11.5–22.2)^c^	$386,676
Under 19 s								
Lopez et al. [68]	3	194	15.5 (5.0–47.9)	$32,223	1	260	3.8 (0.5–27.3)	$10,741
Amateur/elite candidate								
Lopez et al. [67]	7	1111	6.3 (3.0–13.2)^b^	$75,187	14	1480	9.5 (5.6–16.0)	$150,374
Pooled rugby 7 s total	39	2993	13.0 (9.5–17.8)	$418,899	51	3990	12.8 (9.7–16.8)	$547,791
** Pooled total**	**41**	**3580**	**11.5 (8.4–15.6)**	**$440,381**	**53**	**4503**	**11.8 (9.0–15.4)**	**$569,273**

*n* number, *IR* incidence rate, *CI* confidence interval, *AEs* athlete exposures

^a^Costs are reported in New Zealand dollars (NZ$) based on the average cost per concussion claim [55]

^b^Significant difference (*p* < 0.05) vs. Rugby 7 s Elite 8 season

^c^Significant difference (*p* < 0.05) vs. World Cup level

^d^Significant difference (*p* < 0.05) vs. Amateur/Elite candidate

Five studies [67, 68, 70–72] reported injury burden (see Table 3). At the amateur level of rugby 15 s match participation [70], the concussive injury burden was 28.9 ± 3.7 days. For rugby 7 s, the concussive injury burden ranged from 36.7 (20.8–52.6) days [67] to 37.0 (19.7–54.3) days [68] and 9 days per 1000 player hours [72]. For amateur women’s rugby 15 s training [71], the concussive injury burden was 30 days.

**Table 3:** Injury burden reported for women’s rugby 15 s and rugby 7 s studies, by mean days lost and 95% confidence interval or standard deviation

	Forwards	Backs	Total
	[mean (95% CI)]	[mean (95% CI)]	[mean (95% CI)/SD]
*Rugby 15 s match*			
Amateur			
King et al. [70]			28.9 ± 3.7
*Rugby 7 s*			
Amateur			
Lopez et al. [67]	52.3 (3.7–101.0)	29.5 (15.1–43.8)	36.7 (20.8–52.6)
Elite			
Lopez et al. [68]	24	41.3 (21.3–61.4)	37.0 (19.7–54.3)
*Rugby 15 s training*			
Amateur			
King et al. [71]	–	–	30.0
**Pooled**	**38.2 ± 20.0**	**35.4 ± 8.3**	**33.2 ± 4.3**

*CI* confidence interval, *SD* standard deviation

## Discussion

The aim of this review was to examine the incidence of concussion in rugby union and rugby league match play and training, across all levels of play for women. While recent reviews [3, 103] have reported concussion incidence in rugby league for men, no pooled analysis for women has previously been undertaken. The current pooled analysis incorporates both match and training concussion injuries at levels of participation from collegiate to world championships.

On review of the studies for inclusion, three studies [67, 68, 99] reporting on rugby 7 s were identified as reporting similar data, and some values could not be calculated given the data provided. The lead author was contacted for clarification of the results provided in the articles. As a result of the replies from the author, only one study [68] was included in the analysis as this study covered the other two previously published studies [67, 99]. The number of concussions reported were differentiated into time loss (*n* = 15) and medical attention (*n* = 6) concussion injuries, with a definition of > 1 day absent for any concussion, not just seen by medical staff for medical attention (personal email dated 7 August 2020). It is not clear whether the six concussions that were assessed and did not result in time loss were suspected concussions that did not go on to have a medically confirmed diagnosis or if these were confirmed concussions that returned to full participation on the same day of injury.

### Injury-Related Costs for Concussion

In a review of the associated costs of sport-related concussion in five sports in New Zealand over a 10-year period (2001–2011) [55], there were 1330 Accident Compensation Corporation (ACC) moderate-to-severe injury claims (MSCs) costing a total of $13,039,416, or $9804 per claim. The costs for the concussions recorded in this study [55] were classified as MSCs, meaning the injury required additional financial support for treatment, loss of earnings and related medical costs [104, 105]. This is not an indication of the injury severity but gives an indication of the impact that the injury has on the person’s life. The costs do not include presentations to a hospital as these are covered by bulk payments made by ACC to the hospitals. Rugby union recorded the highest total costs ($6,252,870) but rugby league recorded the highest mean cost per claim ($25,545). When female claims and costs were separated, female rugby union concussion cost a total of $608,215, with a mean cost of $9078, while female rugby league cost less at $121,732 but had a higher mean cost per claim ($10,144). When adjusted for inflation (Reserve Bank inflation adjustor; https://www.rbnz.govt.nz/monetary-policy/inflation-calculator), the costs were $719,645 ($10,741 per claim) for rugby union and $114,034 ($12,002 per claim) for rugby league. However, these costs were only for those concussive injuries that were seen more than once for further management and included additional financial support for treatment, loss of earnings and related medical costs [104, 105]. Given these average costs, the concussions reported in this review would equate to approximately $2,358,584 (rugby union: $2,298,574; rugby league $60,010), with concussions to backs costing more ($569,273 vs. $440,381) when compared with forwards.

The differences in the costs between rugby league and rugby union may be related to the period when the study was conducted and how concussion was identified and managed. With the increased awareness of the effects of concussion, players may be seeking medical assistance earlier and this may be reflected in the costs reported. Ongoing research reporting on the costs of concussions in rugby union and rugby league is warranted to identify the costs of these injuries.

### Concussion Injury Burden

The finding that the mean injury burden (days lost) varied by participation level may be more reflective of the level of medical support provided, with female participation having minimal or no medical support available at the sideline, at all participation levels. Of the studies included in this review, the mean injury burden (days lost) for concussions, when pooled together, was 33 days, which was similar to a previous study [30] where the majority (77%) of concussions (male and female) took 28 days to recover and females had a 43–48% longer recovery time when compared with males. This is considerably longer than the 7- to 10-day expected timeframe outlined in the Concussion in Sport Consensus (CISC) statement for 80–90% of all concussions [106–108]. These longer recovery times indicate that the CISC guidance may need to be updated, include a breadth of sport-related concussion studies, and consider sex-specific recommendations. Further research is warranted to better understand concussion recovery in female athletes and the identification of tailored treatments for female concussions [40].

### Male Versus Female Differences in Concussion

In a recent study [109] reporting on differences between male and female student athletes, the incidence of concussion was notably higher in female participants in sex-comparable sports (i.e., male and female teams play under the same rules and with the same equipment) and these concussions were more severe when compared with male participants. However, there is a paucity of studies reporting women’s rugby 15 s, rugby 7 s and rugby league when compared with male participation in these sporting codes. In examining symptom cluster discrepancies between male and female athletes, both at baseline and post-concussive event [34, 110, 111], it was reported that female athletes experienced more symptoms than male athletes. In a recent study [109], it was identified that females had notably higher scores in three of the concussion symptom clusters (migraine, cognitive and neuropsychiatric) in the acute concussion phase and at follow-up when compared with males. The cognitive and migraine clusters are classic symptoms of concussion [112], whereas the neuropsychiatric symptom cluster (irritability, nervousness, sadness, feeling more emotional) is less traditional and has a reported insidious effect on daily living and recovery [113]. Females have been reported to have a higher risk of mood disturbances after a concussive injury [109]. It has been recommended that sex-based considerations for managing concussion risk may be necessary to prevent further widening of the gap in concussion incidence between male and female sports participants [109].

### Cause of Injury

In a study reporting on male and female athletes between the ages of 5 and 19 years [91], rugby union accounted for 5.6% (*n* = 721) of brain injuries. The primary mechanisms for a concussion occurring in females in rugby union were being tackled or tackling another player (51.7%), head-to-head collisions (7.3%) and head-to-knee collisions (5.1%). This was similar to another study reporting on women’s amateur rugby 15 s [96], where the majority (88%) of concussions occurred in the tackle (tackler: 11%; ball carrier: 77%) and only 11% of the concussions occurred in the ruck situation, and the exact mechanism of injury was not reported. One study [14] comparing both male and female rugby 15 s participants reported an incidence of 2.2 per 1000 match hours and 1.6 per 1000 match hours for males and females, respectively. The results of this study were consistent with another study reporting on the injury risk associated with tackling in rugby 15 s [114].

### Injury Definition

As previously identified, what constitutes a reliable and accurate injury definition remains a contentious issue [58, 59] and this can be seen by the different types of injury definitions reported in this review. Injury definition and exposure heterogeneity can limit interstudy comparability, and this was evident with three studies not reporting exposure rates and three studies reporting a different exposure rate (AE). Although more than half of the included studies [10, 11, 13–15, 66–68, 70, 71] utilised an inclusive-type injury definition, this was evenly split, with five studies reporting an all-inclusive [10, 13–15, 68] injury definition and five studies reporting a fully-inclusive [11, 66, 67, 70, 71] injury definition. The use of these definitions is similar; however the fully-inclusive definition reports on missed match and training time and excludes injuries that require assistance from medical personnel [57, 60]. This is problematic as any injury that does not result in a missed match and occurs in a limited training session may be declared recovered and not recorded as an injury [115]. Missed-match definitions also depend on the duration between matches [115], with some studies reporting on tournaments where matches may occur more than once in a 7-day period or more than one match a day for consecutive days.

### Limitations

As identified, there is a paucity of published literature reporting on female participation in both rugby union and rugby league injury epidemiology. Areas such as interstudy comparisons are limited by the different methodological approaches and definitions utilised as identified in this study. The publication of the consensus for data collection in rugby union studies [116] and the more recent community-based injury surveillance in rugby union [117] provides for a standardised approach that should be considered for future rugby union and rugby league studies. This will assist with the identification of the injury site, type, location, severity and mechanism of injury that occurs within female rugby union and rugby league.

Typically, most of the studies included in this review were undertaken over a single season or competition, limiting the identification of concussion injury trends, and future longitudinal studies should be encouraged in female rugby union and rugby league activities. There is an increasing awareness of the issue of concussion in rugby union and rugby league and a standardised approach to the assessment of this injury in women participants is necessary in terms of assessment tools, concussion definition and recovery management. Although the data collection methods appear to be similar for males and females, there are differences in terms of injury risk and recovery when comparing the sexes [118, 119]. As such, some aspects unique to females may be important for future research being undertaken, such as the relationship between the phase of the menstrual cycle and the incidence of concussive injuries [38, 120–123]. This information would be beneficial towards the development of tailored injury management for female participants [40].

Other limitation considerations identified in conducting this review were that the included studies ranged from 1999 to 2021, and much has occurred in regard to the definition, recognition, and medical management of concussions, including return to play, over that period of time. This may be reflected in the differences in the injury severity and injury burden recorded for concussions over the duration of the analysis. In addition, the included studies also span different levels of competition (amateur to professional) as well as national populations and may have different periods between match events. These factors may impact how concussion care is delivered, particularly when one considers a professional athlete versus an amateur/community athlete. The differences in medical care availability when comparing amateur and professional athletes or across nations and their healthcare systems are factors to be considered and these are difficult to control for. In addition, the differences in access to medical care across the different healthcare systems may impact upon the return-to-sport duration after a concussion has occurred.

## Conclusions

Our pooled analysis provided combined estimates of concussion injuries for training and games within rugby league and rugby union and showed differences in concussion injury rates and possible associated costs at several levels in the game for women. It has been recommended that sex-based considerations for managing concussion risk may be necessary to prevent further widening of the gap in concussion incidence between male and female sports participants. The pooled mean days lost because of concussions was 33 days. As this was considerably longer than the 7- to 10-day expected timeframe outlined in the CISC statement, these guidelines need to be updated to include sex-specific differences.

## Supplementary Information

Below is the link to the electronic supplementary material.

Supplementary file1 (DOCX 31 kb)

## Declarations

### Data availability

Data sharing is not applicable to this article as no datasets were generated or analysed during the current study.
