# Supplementary material for: The Incidence, Cost, and Burden of Concussion in Women’s Rugby League and Rugby Union: A Systematic Review and Pooled Analysis
Source: Sports Med. 2022 Feb 3;52(8):1751–64. doi: 10.1007/s40279-022-01645-8 (PMC9325800; doi:10.1007/s40279-022-01645-8)
Supplement: Supplementary file 1 — Supplementary file1 (DOCX 31 kb) [file 40279_2022_1645_MOESM1_ESM.docx]

**Online Resource**

**Table S1**: Meta-Analyses and Systematic Reviews of Observational Studies (MOOSE)^[49]^ assessment of reviewed publications included in the pooled analysis.

| Study | Year | Prospective | Groups comparable  on confounding factors | Blinded outcome | Long enough  follow-up | Exposure response  measured | Appropriate  statistics | Overall quality (max =6) |
| --- | --- | --- | --- | --- | --- | --- | --- | --- |
| Carson et al.^[13]^ | 1999 | Yes | Yes | No | Yes | Yes | Yes | 5 |
| King et al.^[19]^ | 2007 | Yes | Yes | No | Yes | Yes | Yes | 5 |
| Schick et al.^[10]^ | 2008 | Yes | Yes | No | Yes | Yes | Yes | 5 |
| Kerr et al.^[14]^ | 2008 | Yes | Yes | No | Yes | Yes | Yes | 5 |
| Collins et al.^[16]^ | 2008 | No | Yes | No | Yes | Yes | Yes | 4 |
| Taylor et al.^[11]^ | 2011 | Yes | Yes | No | Yes | Yes | Yes | 5 |
| Peck et al.^[15]^ | 2013 | No | Yes | No | Yes | Yes | Yes | 4 |
| Lopez et al.^[67]^ | 2016 | Yes | Yes | No | Yes | Yes | Yes | 5 |
| Fuller et al.^[66]^ | 2017 | Yes | Yes | No | Yes | Yes | Yes | 5 |
| King et al.^[69]^ | 2018 | Yes | Yes | No | Yes | Yes | Yes | 5 |
| Toohey et al.^[72]^ | 2019 | Yes | Yes | No | Yes | Yes | Yes | 5 |
| King et al.^[70]^ | 2020 | Yes | Yes | No | Yes | Yes | Yes | 5 |
| Langevin et al.^[73]^ | 2020 | Yes | Yes | No | Yes | Yes | Yes | 5 |
| Lopez et al.^[68]^ | 2020 | Yes | Yes | No | Yes | Yes | Yes | 5 |
| Yeomans et al.^[74]^ | 2021 | Yes | Yes | No | Yes | No | Yes | 4 |
| King et al.^[71]^ | 2021 | Yes | Yes | No | Yes | Yes | Yes | 5 |
